# Intersystem crossing-branched excited-state intramolecular proton transfer for o-nitrophenol: An ab initio on-the-fly nonadiabatic molecular dynamic simulation

**DOI:** 10.1038/srep26768

**Published:** 2016-05-25

**Authors:** Chao Xu, Le Yu, Chaoyuan Zhu, Jianguo Yu, Zexing Cao

**Affiliations:** 1 College of Chemistry, Beijing Normal University, Beijing 100875, P. R. China; 2 Institute of Molecular Science, Department of Applied Chemistry and Center for Interdisciplinary Molecular Science, National Chiao Tung University, Hsinchu 30010, Taiwan; 3 Key Laboratory of Synthetic and Natural Functional Molecule Chemistry of Ministry of Education, The College of Chemistry & Materials Science, Shaanxi key Laboratory of Physico-Inorganic Chemistry, Northwest University, Xi’an 710069, P. R. China; 4 State Key Laboratory for Physical Chemistry of Solid Surfaces and Fujian Provincial Key Lab of Theoretical and Computational Chemistry, College of Chemistry and Chemical Engineering, Xiamen University, Xiamen 361005, P. R. China

## Abstract

The 6SA-CASSCF(10, 10)/6-31G (d, p) quantum chemistry method has been applied to perform on-the-fly trajectory surface hopping simulation with global switching algorithm and to explore excited-state intramolecular proton transfer reactions for the o-nitrophenol molecule within low-lying electronic singlet states (S_0_ and S_1_) and triplet states (T_1_ and T_2_). The decisive photoisomerization mechanisms of *o*-nitrophenol upon S_1_ excitation are found by three intersystem crossings and one conical intersection between two triplet states, in which T_1_ state plays an essential role. The present simulation shows branch ratios and timescales of three key processes via T_1_ state, non-hydrogen transfer with ratio 48% and timescale 300 fs, the tunneling hydrogen transfer with ratios 36% and timescale 10 ps, and the direct hydrogen transfer with ratios 13% and timescale 40 fs. The present simulated timescales might be close to low limit of the recent experiment results.

The excited-state intramolecular proton transfer (ESIPT) reaction is considered to be one of the most fundamental and important processes in chemistry, material and biology^1^,^2^,^3^,^4^,^5^,^6^,^7^,^8^,^9^,^10^,^11^,^12^. The ESIPT reactions have been studied from both ab initio quantum chemistry calculations^13^,^14^,^15^,^16^,^17^ and trajectory-based nonadiabatic molecular dynamic simulations^18^,^19^,^20^,^21^,^22^. The most of the ESIPT processes have been found to be taken place at a subpicosecond time scale^20^,^21^,^22^. The photo-excited molecule from electronic ground singlet state (S_0_) to the excited singlet states can undergo rapid internal conversion via conical intersections (CI) to the lower singlet states as well as intersystem crossings (ISC) via spin-orbital couplings (SOC) to the triplet states. By means of the radiative or nonradiative interaction with target molecule, the redistribution of electron density is essential for the transfer of a hydroxyl (or amino) proton to an oxygen or nitrogen acceptor atom on the excited states within a hydrogen bond already formed in the electronic ground state. Subsequently, the excited state molecule can decay to the ground state accompanied with a reverse proton transfer.

Fundamental mechanistic insight into photoinduced ESIPT reaction can be understood through studying prototypical representative of nitroaromatics *o*-nitrophenol molecule. This is because that *o*-nitrophenol owns two functional groups, namely nitro and the adjacent hydroxyl groups which show strong intramolecular hydrogen bond. Thus, the aci-nitrophenol can be formed via the initial intramolecular hydrogen transfer from hydroxyl group to nitro group. Differing from the other aromatic systems, the experimental and theoretical studies demonstrate peculiar behavior of the photoinduced decay process for nitrated aromatic compounds. The tautomeric aci-nitrophenol isomers via photoisomerization has been confirmed by infrared spectroscopy in low-temperature argon matrices^23^ and by laser-induced-fluorescence^24^. The rich intersystem crossing network makes *o*-nitrophenol from the first excited singlet state decaying to the triplet states rapidly, and the process occurs in the femtoseconds to a few picoseconds time scale dependent on the intermediate states^25^,^26^,^27^. The early experiment showed that time scale decaying to the first excited triplet state is lower than 50 ps by measuring the UV excitation of *o*-nitrophenol in benzene solvent^28^. However, the recent experiment showed the existence of unstable aci-nitrophenol isomers and ultrafast ISC to the triplet manifold on a subpicosecond time scale by both femtosecond transient absorption spectroscopy in solution and time-resolved photoelectron spectroscopy in the gas phase^29^.

Up to now, neither fluorescence nor phosphorescence has been reported for *o*-nitrophenol and this indicates the existence of pure ultrafast radiationless decay process^29^. Therefore, an ab initio on-the-fly nonadiabatic molecular dynamics simulation is necessary to be performed to quantitatively analyze ESIPT process of o-nitrophenol via internal conversion and intersystem crossing network. This is motivation of the present study. By using ab initio quantum chemistry method at 6SA-CASSCF(10, 10)/6-31G (d, p) and MRCI/cc-pVDZ level, we previously optimized geometries for all isomers and transition states on two low-lying singlet (S_0_ and S_1_) and two low-lying triplet (T_1_ and T_2_) electronic states and we computed all four-state potential energy profiles along ESIPT coordinates^30^. We confirmed the existence of unstable aci-nitrophenol isomers as observed in the early studies^23^,^24^,^29^,^30^. We found total five ISC zones with SOCs at ~10 and ~40 wavenumbers. By performing nonadiabatic molecular dynamics simulation in the present study, we actually found one new S_0_/T_1_ ISC which governs decay process for non hydrogen transfer. The previous computational and experimental study has proposed two possible relaxation pathways^29^; one is via the ISC following (O)H-O(NO) stretch and the other is via the S_0_/S_1_ CI after overcoming potential energy barrier induced by hybrid torsion of HONO group and (O)H-O(NO) stretch. Actually, the new S_0_/T_1_ ISC is located near Frank-Condon region while S_0_/S_1_ CI is located in configuration after hydrogen transfer. The present simulation has shown that the new S_0_/T_1_ ISC is very effective pathway and there is almost no trajectory going via S_0_/S_1_ CI. We can expect that there must have strong intersystem crossing-branched ESIPT. Besides, the existence the certain potential barriers on S_1_ and T_1_ states along ESIPT coordinate can add tunneling effects as well. The present study should reveal entire picture of photoisomerization reaction and the photo-decay mechanisms including ESIPT for o nitrophenol molecule.

Trajectory-based ab initio nonadiabatic molecular dynamics simulations have been successfully applied to photoisomerization and photoreaction processes involving intersystem crossings and conical intersections^31^,^32^,^33^,^34^,^35^,^36^,^37^,^38^,^39^,^40^,^41^,^42^,^43^,^44^,^45^,^46^,^47^,^48^. Recently, within Tully’s fewest switching algorithm in diabatic representation dynamic simulations have been carried out for intersystem crossings^43^,^44^,^45^,^46^,^47^,^48^. We have developed trajectory-based ab initio nonadiabatic molecular dynamics simulations without involving calculation for nonadiabatic couplings^37^, and it has been applied for multiple-state azobenzene photoisomerization via conical intersections^38^. Now we extend our analytically global switching probability method to include intersystem crossing with spin-orbital couplings. Trajectories are calculated on on-the-fly ab initio singlet or triplet potential energy surfaces and then by applying global switching algorithm we treat trajectory surface hopping in a unified way for internal conversion and intersystem crossing processes. Intersystem-crossing dynamic simulation with global switching surface hopping algorithm is not really new with Landau-Zener switching probability; the spin-diabatic and spin-adiabatic dynamic simulations^31^ were performed for the model system of spin-orbital couplings that are used as diabatic coupling parameter in Landau-Zener formula, and moreover good agreement with experimental observation for the triplet to singlet branching ratio has been achieved with Landau-Zener formula for performing real dynamic simulation of O(^3^P) + ethylene^32^. We utilize improved Landau-Zener formula, namely Zhu-Nakamura to treat intersystem-crossing dynamic simulation in the present report. Moreover, tunneling effects are treated by one-dimensional semilcassical method along ESIPT coordinate^49^.

## Results

### Global switching algorithm

Global switching algorithm makes trajectory hopping at the time *t* where 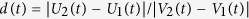 reaches local maximum (*U*_2_ and *U*_1_ are adjacent two adiabatic potential energy surfaces, while *V*_2_ and *V*_1_ are corresponding two diabatic potential energy surfaces). Analytical switching probability can be generally expressed in terms of *d*(*t*) as^50^


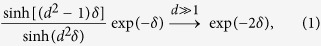


in which *δ* is estimated from two potential energy surfaces and its gradients at hopping spot along on-the-fly running trajectory. In the case of *d* 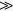 1, it basically goes to Landau-Zener or improved version Zhu-Nakamura formula^51^,^52^,


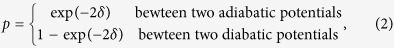


where


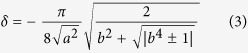


in which the effective coupling parameter *a*^2^ and effective collision energy *b*^2^ are given by


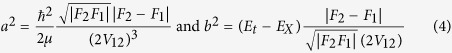


where *F*_1_ and *F*_2_ are forces on two diabatic potential energy surfaces, *V*_12_ is diabatic coupling, μ is reduced mass and *E*_X_ is energy at crossing point and *E*_t_ is potential energy plus kinetic energy component in direction of hopping direction. All those quantities in equation 4 are calculated along on-the-fly running trajectory at hopping spot^37^ and its details are also given in the Supplementary Note 1. The hopping direction in the present theory is defined based on sort of the local modes and this agrees with the normal modes constructed from the regularized diabatic states^53^. Global switching algorithm has been extensively compared with the Tully’s fewest switching algorithm^37^,^40^ and two algorithms are basically similar for highly averaged quantities like quantum yields and lifetimes. In the present on-the-fly simulation, along three consecutive time steps we detect minimum energy separation between two singlet-states (S_1_ and S_0_) or two triplet-states (T_2_ and T_1_) and this gap is considered as diabatic coupling 2*V*_12_ in equation 4. In this case, the switching is considered as in between two adiabatic potential energy surfaces. On the other hand, we detect the local maximum *d(t)* between the singlet and triplet states. The diabatic coupling is considered as spin-orbital coupling as *V*_12_ = SOC in equation 4 as proposed in ref. 43., and in fact, singlet state *V*_1_ and triplet state *V*_2_ can be transformed into adiabatic representation according to


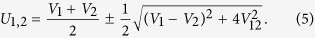


However, in this case, we can do the switching directly between the singlet and triplet states as the spin-orbital coupling is known.

### Intersystem crossings with ab initio dynamics

**Figure 1:**
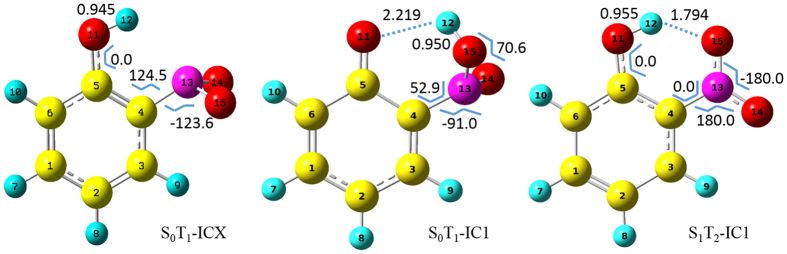
Key geometries for three active intersystem crossings in trajectory surface hopping dynamics (also for atomic numbering).

**Figure 2:**
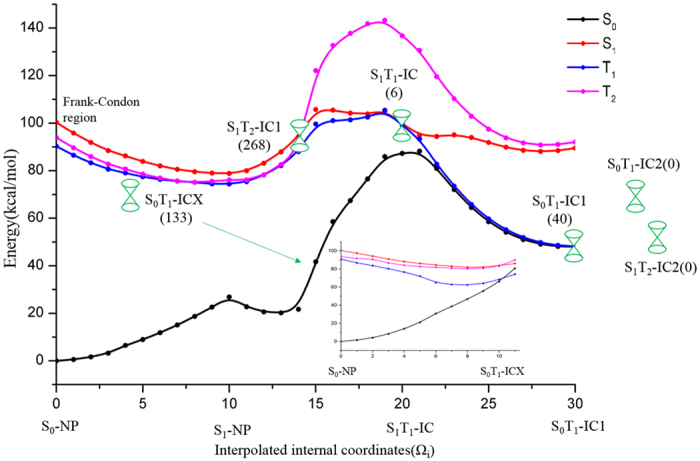
Potential profiles along ESIPT reaction coordinates. Four intersystem coursings (S_1_T_2_-IC1(SOC = 10 cm^−1^), S_1_T_1_-IC(SOC = 40 cm^−1^), S_0_T_1_-IC1(SOC = 40 cm^−1^) and S_0_T_1_-ICX(SOC = 40 cm^−1^)) are approximately along this path and the other two (S_0_T_1_-IC2(SOC = 40 cm^−1^) and S_1_T_2_-IC2(SOC = 10 cm^−1^)) are far way. Number in parentheses associated with each intersystem crossing represents trajectories going via it. The small widow stands for potential energy profiles for newly found S_0_T_1_-ICX. The Cartesian coordinates of six ICs are summarized in the Supporting Information.

We have previously performed ab initio quantum chemistry calculations at 6SA-CASSCF(10, 10)/6-31G (d, p) level and have compared with MRCI/cc-pVDZ energy corrections for two singlet states (S_0_ and S_1_) and two triplet states (T_1_ and T_2_). It is well-known that the CASSCF method is lack of dynamic correlation. Therefore, we carried out energy correction by multi-reference configuration interaction (MRCI) calculation at CASSCF optimized geometries. Both vertical excitation energies (see Fig. S1(a) at CASSCF and Fig. 1S(b) at MRCI given in Supplementary Note 2) and adiabatic energies (see Fig. S2(a) at CASSCF and Fig. 2S(b) at MRCI given in Supplementary Note 2) at all key geometries show the same energy sequences. Relative energy differences between CASSCF and MRCI are mostly smaller than 0.1 eV, especially for energy gaps at six intersystem crossings and one conical intersection being even smaller (see Table S1). Therefore, we think that the present on-the-fly dynamical simulation based on CASSCF level should present the almost same results as it is based on MRCI level. We have confirmed 6SA-CASSCF(10, 10)/6-31G (d, p) is suitable choice for the present dynamic simulation.

We found there are two intersystem crossings between S_1_ and T_2_ (S_1_T_2_-IC1 and S_1_T_2_-IC2) around which SOC can be almost considered as constant 10 cm^−1^, one between S_1_ and T_1_ (S_1_T_1_-IC) around which SOC is 40 cm^−1^, three between S_0_ and T_1_ (S_0_T_1_-IC1, S_0_T_1_-IC2 and S_0_T_1_-ICX) around which SOCs are close to 40 cm^−1^. The S_0_T_1_-ICX is newly found in the present study by trajectory surface hopping dynamic simulation, and the other five were optimized previously^30^. Furthermore, the present dynamic simulation shows that only three of six are active for ESIPT reaction, namely S_1_T_2_-IC1, S_0_T_1_-ICX, and S_0_T_1_-IC1 as shown in Fig. 1. The S_1_T_1_-IC is less active, and S_1_T_2_-IC2 and S_0_T_1_-IC2 are not active at all (key geometries and Cartesian coordinates for all six intersystem crossings are given in Supplementary Note 2 and Note 4). Figure 1 shows that the dihedral angels C_4_C_5_O_11_H_12_ are zero for S_0_T_1_-ICX and S_1_T_2_-IC1, and the O_11_H_12_ bond lengths are 0.945 Å and 0.955 Å, respectively. However, unlike to the planar geometry of S_1_T_2_-IC1, the nitro group of S_0_T_1_-ICX and S_0_T_1_-IC1 show a similarity although the H_12_ bonded to O_11_ in S_0_T_1_-ICX instead of O_15_ in S_0_T_1_-IC1. The nitro group of them is out of the aromatic skeleton. Those geometry differences essentially govern the three intersystem crossings undergoing distinct ESIPT reaction pathways. Interestingly, the structure of S_0_T_1_-ICX is close Franck-Condon geometry and the structure of S_0_S_1_-CI is close to S_0_T_1_-IC1 geometry (see Fig. S3 given in Supplementary Note 2). The S_0_T_1_-ICX is responsible for relaxing pathway of non-hydrogen transfer, while the S_0_T_1_-IC1 responsible for relaxing pathway of after-hydrogen transfer.

The initial condition of trajectories is started from Franck-Condon region of *o*-nitrophenol. We perform frequency calculation at the ground state equilibrium geometry of S_0_ to obtain the normal mode coordinates. Initial normal-mode coordinates and velocities of trajectory are selected according to the Wigner distribution on S_0_ state. Finally, these initial normal-mode coordinates and velocities are converted into Cartesian coordinates and velocities plus vertical excitation energy to excited S_1_ state. The thermal kinetic energy with T = 300K is added to all sampling trajectories with randomly distributing into initial Wigner velocities. However, such equally distributed initial conditions are not suitable for stimulating ESIPT reaction, there are the certain vibronic modes enhanced more than the others as molecule absorbed light to be excited to S_1_ state. We found that four normal modes (O_11_H_12_ stretch (4172 cm^−1^), C_5_O_11_H_12_ bend (1473 cm^−1^), and two C_5_O_11_H_12_-C_4_N_13_O_15_ scissor (408cm^−1^ and 310 cm^−1^)) involved in the ESIPT reaction path have stronger vibronic couplings than the others. Therefore, we added extra kinetic energy (equivalent to T = 500 K) to these four normal modes. Along an on-the-fly running trajectory, the nuclear coordinates and velocities in Cartesian coordinates are propagated by numerically integrating the Newtonian equation of equation of motion with the velocity-Verlet method^54^. We determine the minimum potential energy gap between S_1_ and S_0_ states, and between T_1_ and T_2_ states at the conical intersection zones, and determine maximum *d* (t) between singlet and triplet states at intersystem crossing zones, at which we compute the effective coupling parameter *a*^2^ and the effective collision energy *b*^2^. We found SOC varies very slowly at intersystem crossing zones, so that we choose constants accordingly 10 and ~40 cm^−1^ in simulation (when the energy gap is smaller than 0.25 eV, we start to check attempted trajectory surface hopping). We have run several expensive on-the-fly SOC trajectories in comparison with fixed SOC trajectories and results show small difference. We have checked for four active intersystem crossings, relative spin-orbital coupling variations (δSOC/SOC) are about less than 2% around crossing zones in which trajectory hops take place. This fixed SOC technique was also utilized for performing real dynamic simulation of O(^3^P) + ethylene^32^.

Due to the present dynamics involving OH stretch vibration (over 3500 cm^−1^) along ESIPT reaction path, we made test runs with time steps of 0.05 fs, 0.1 fs and 0.15 fs, and finally we set up the 0.1 fs time step from the beginning to the end for the entire dynamic simulation. The dynamics simulation time is set up as 500 fs and as we increase up to 1000 fs, there is no notable difference. All the quantum chemical calculations of on-the-fly potential energy surfaces and its gradients are carried out at 6SA-CASSCF(10, 10)/6-31G (d, p) level by using the quantum chemistry package MOLPRO 2009.1.^55^ and the dynamic simulation is carried by our own code.

### On-the-fly trajectory analysis

**Figure 3:**
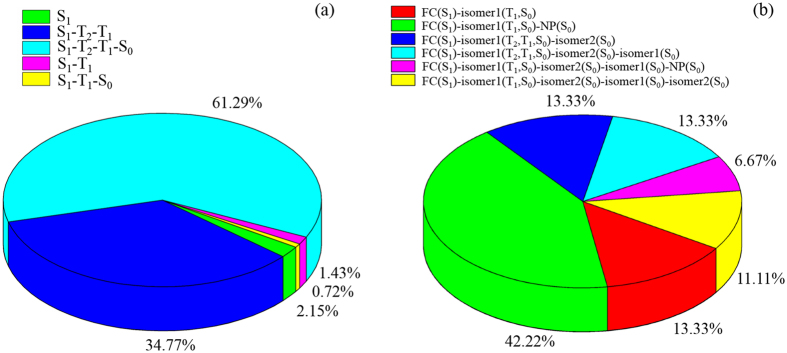
(**a**) Distribution of multiple nonadiabatic transition pathways among the lowest four electronic states (S_1_, T_2_, T_1_ and S_0_). (**b**) Detailed distribution of trajectories passing through the isomers of the *o*-nitrophenol and aci-nitrophenol (aci-isomer1 and aci-isomer2).

We have run total 280 trajectories and the outcomes show that 6 stay on S_1_ state regarded as resonance, 6 go via S_1_T_1_-IC as direct hydrogen transfer via T_1_ state, and 268 go via S_1_T_2_-IC1 initially as shown in Fig. 2 (this is basically Fig. 7 in ref. 30). After going via S_1_T_2_-IC1, 265 trajectories hop to T_1_ states via conical intersection between T_1_ and T_2_ states, and 3 go direct hydrogen transfer via T_2_ state. Further three bifurcations take place for the 265 trajectories on T_1_ state, 101 regarded as resonance on T_1_ state, 133 going via S_0_T_1_-ICX as non-hydrogen transfer, and 31 as direct hydrogen transfer via T_1_ state. This is overview how trajectories decay through competing nonadiabatic pathways in the singlet and triplet excited-state manifold. The percentage distribution of various nonadiabatic transition pathways is shown in Fig. 3a. The present ab initio dynamic simulation shows that the S_1_ → T_2_ → T_1_→ S_0_ (61.29%) and S_1_ → T_2_ → T_1_ (34.77%) are the dominant processes, while the S_1_ → T_1_→ S_0_ (0.72%) and S_1_ → T_1_ (1.43%) are kind of rare cases. Initially starting from Frank-Condon region on S_1_ state, the majority sampling trajectories run in the electronic configuration where three excited states (S_1_, T_2_ and T_1_) are energetically close together, T_2_ state always keeps in between the S_1_ and T_1_ states for a while. On the other hand, the present ab initio dynamic simulation confirms that intersystem crossing zone between the S_1_ and T_2_ states with small SOC (10 cm^−1^) are much wider than intersystem crossing zone between the S_1_ and T_1_ states with large SOC (40 cm^−1^). Therefore, without performing ab initio dynamic simulation, we could not realize that S_1_→T_2_ intersystem crossing (ISC-S_1_T_2_) channel is paramount relaxing pathway. Actually, similar situation is found that the efficient ultrafast ISC dynamic can still be taken place with a rather small spin-orbit coupling due to the counterbalance mechanism^56^,^57^. Further detailed distribution is shown in Fig. 3b for the sampling trajectories passing through the isomerization of the *o*-nitrophenol and aci-nitrophenol. In the present simulations, we defined the aci-nitrophenol isomers as aci-isomer1 and aci-isomer2, respectively. For aci-isomer1, it still exhibits intramolecular hydrogen bond with ortho oxygen atom of benzene and while for aci-isomer2, the hydrogen rotates with nitro group and far away from the ortho oxygen atom. Diverse distributions are shown, for instance, that *o*-nitrophenol overcomes barrier of hydrogen transfer and reaches the aci-isomer1 (13.33%), after getting over the hydrogen transfer barrier it passes to aci-isomer1 and then back to *o*-nitrophenol (42.22%), the *o*-nitrophenol converts to aci-isomer1 and then continue to produce aci-isomer2 with the inversion of O_15_H_12_ (13.33%), the o-nitrophenol changes to aci-isomer1and then regenerated again after going via aci-isomer2 (13.33%), and so on. Starting from Frank-Condon region on S_1_ state, among 280 sampling trajectories there are 3, 37, and 5 trajectories undergoing direct hydrogen transfer on T_2_, T_1_ and S_0_ states, respectively. However there are 6 and 101 trajectories undergoing tunneling hydrogen transfer on S_1_ and T_1_ states, respectively. Although direct hydrogen transfer on T_2_ is rare case, it presents a new perspective for general ESIPT reactions. In most of situation, the T_2_ state looks more like a “hub”, trajectories stop on it for a moment and then quickly decay to the reactive T_1_ state where is major channel for direct hydrogen transfer. The barrier for hydrogen transfer on T_1_ state is lower than that of on S_1_ state as shown in Fig. 2. On the other hand, the present dynamic simulation shows that kinetic energy from the four vibartional normal modes responding for hydrogen transfer easily dissipates to the other vibrational modes on S_1_ state much faster than on T_1_ state. This is part of reason that direct hydrogen transfer does not occur on S_1_ state. Hydrogen transfer has a peculiar property on S_0_ state which differs from that of on T_1_ state, trajectory reaches to S_0_ state via multi-steps continuous hops in a relatively short period of time and then it runs on the ground state, eventually the hydrogen transfer reaction occured and aci-isomer formed. It should be noted that the aci-isomer can undergo backward hydrogen transfer reaction to o-nitrophenol due to very low barrier (nearly barrier less). This is reason that hydrogen transfer occurs on S_0_ state not very often. The newly found intersystem crossing S_0_T_1_-ICX has its geometry close to S_0_-NP, so that 133 out of 280 go via it and those trajectories relax to ground state by non-hydrogen transfer pathway. In brief summary, we conclude that S_0_T_1_-ICX serves as the center player of intersystem crossing for three competing processes, 45 trajectories results in direct hydrogen transfer, 107 in tunneling hydrogen transfer, and 128 in non-hydrogen transfer.

**Figure 4:**
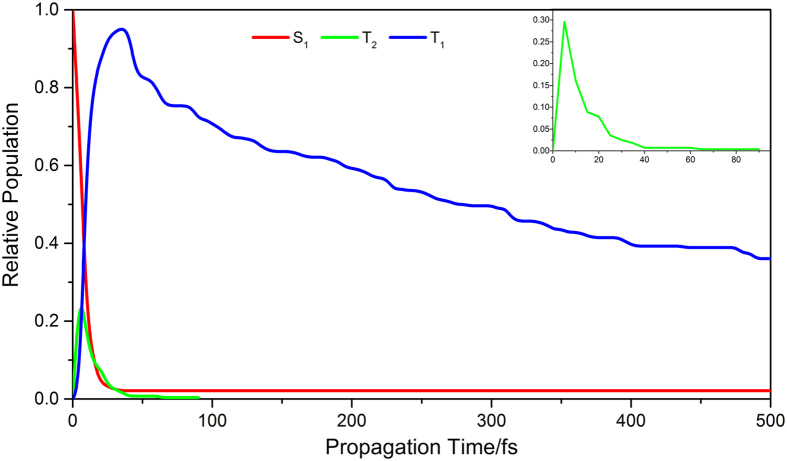
Relative population distribution on the S_1_, T_2_ and T_1_ states as a function of time (small window is for T_2_ state).

Although every trajectory has its own lifetime when surface hopping occurs after photoexcitation, and the final result is estimated by the average assemble of all sampling trajectories. Figure 4 shows average population decay with respect of time from excited states S_1_, T_2_ and T_1_, respectively. A single exponential function curve fitting is applied to calculate average lifetimes on those excited states. The lifetime of S_1_ state was estimated to be about 8 fs as an ultrafast decay process and population is almost diminished after 25 fs. This is understandable in common sense that the first excited state decay more likely undergoes via intersystem crossing rather than vibrational cooling^58^,^59^. The decaying process is also an ultrafast on T_2_ state and the lifetime of T_2_ state is estimated to be 14 fs. The population on T_2_ state is quickly raised first and it reaches maximum at 10 fs and than it almost diminishes after 40 fs. The most of population via T_2_ state transfers very quickly to T_1_ state via conical intersection between T_1_ and T_2_ states. The population on T_1_ state reaches its maximum (∼93%) after 40 fs and the lifetime of T_1_ state is estimated to be around 1000 fs. This is the most likely to contribute to the long-lived excited species that should be observed in both gas and liquid phase because the population on T_1_ state decays very slowly. However, the real lifetimes on excited states from experimental observation might be longer than the present estimation due to some extra kinetic energy being initially put in four vibrational modes which are enhancing hydrogen transfer dynamics.

The triplet T_1_ state plays an essential role for intersystem crossing-branched ESIPT photoisomerization dynamics in which 97% sampling trajectories bifurcate into three major distinct decay channels. The first branch is the non-hydrogen transfer that counts for 47% sampling trajectories, and timescale is about 100 fs to 500 fs (average is about 300 fs). For trajectories running on T_1_ state, once they form out-of plane synchronous motion by O_11_H_12_ vibrating around C_5_O_11_ bond and the nitro group vibrating around C_4_N_13_ bond simultaneously, trajectories can reach S_0_T_1_-ICX to decay to ground S_0_ state. The present simulation shows if this out-of plane synchronous motion form coherent motion before trajectories reach S_0_T_1_-ICX (we checked those trajectories from 500 fs to 1000 fs), they stay on T_1_ state as resonance trajectories. This is the second branch as the tunneling hydrogen transfer that counts for 36% sampling trajectories. We have roughly estimated thermal and microcanonical rate constants of nonadiabatic chemical reaction along ESIPT coordinate as shown in Fig. 2 49,


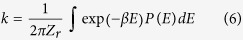


in which *β* is thermal energy (temperature is set up T = 300 K), *Z*_γ_ is reactant partition function and *P*(*E*) is nonadiabatic transmission probability for the tunneling trajectories. The average tunneling timescale estimated from equation 6 is about 10 ps. This tunneling timescale is about the same as tunneling proton transfer reaction for tropolone molecule^60^. The third branch is the direct hydrogen transfer that counts for 13% sampling trajectories, and timescale is from 20 fs to 100 fs (average is about 40 fs). In this case, trajectories can go the direct hydrogen transfer before the out-of plane synchronous motion occurs. Then, the trajectories continue to run on the T_1_ state until they reach S_0_T_1_-IC1 accompanying with O_14_N_13_O_15_H_12_ group out-of-plane rotating and finally hop to S_0_ state.

### Typical trajectories

**Figure 5:**
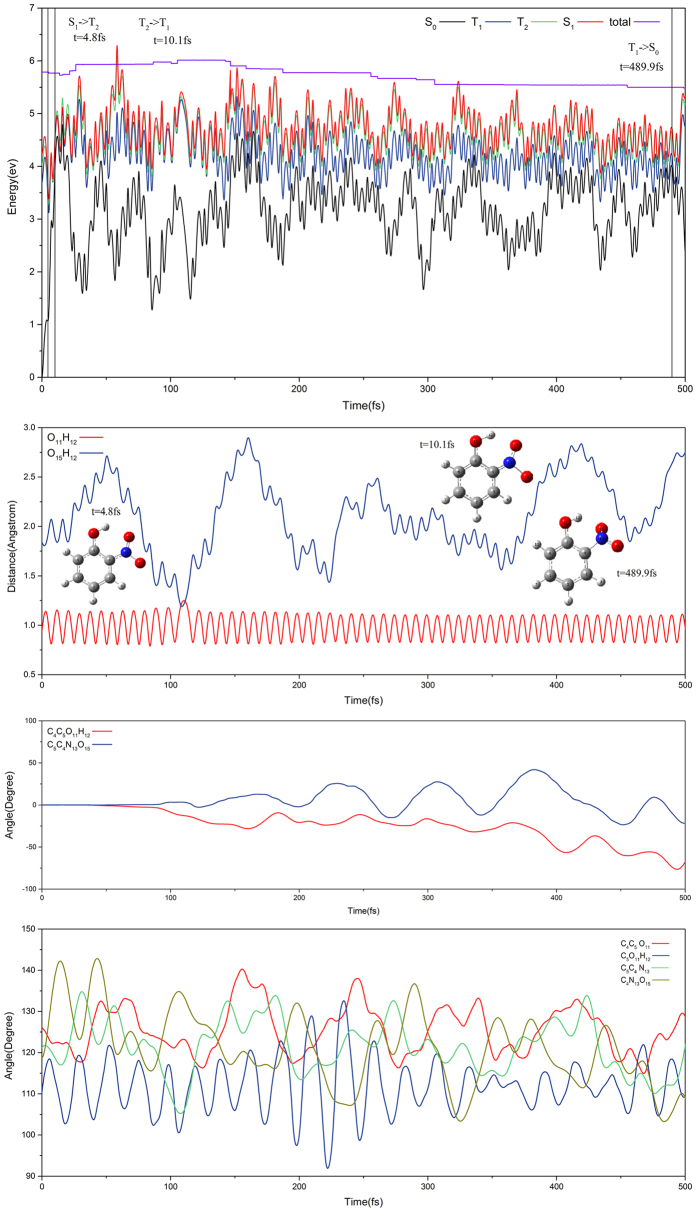
Typical trajectory. The first panel is for potential energy profiles along non-hydrogen transfer channel against the time. The second pane is evolution for bond lengths O_11_H_12_ and O_15_H_12_, the third is for dihedral angles C_4_C_5_O_11_H_12_ and C_5_C_4_N_13_O_15_ and the fourth is for bond angles C_4_C_5_O_11_, C_5_O_11_H_12_, C_5_C_4_N_13_, and C_4_N_13_O_15_.

Figure 5 shows the non-hydrogen transfer for a trajectory that hops to the S_0_ state via S_0_T_1_-ICX at 478.2 fs (this is trajectory staying the longest time on T_1_ state among all non-hydrogen transfer sampling trajectories). Staring from Frank-Condon region on S_1_ state, the trajectory experiences the following consecutive processes, it hops from the S_1_ to T_2_ state via S_1_T_2_-IC1 at the 4.8 fs and from the T_2_ to T_1_ state via conical intersection at the 10.1 fs. During the evolution period of time, O_11_H_12_ and O_15_H_12_ have small and large vibrations, respectively as shown in the second panel of Fig. 5. Two dihedral angles oscillate smoothly while four bond angles oscillate drastically as shown in the third and fourth panels of Fig. 5, respectively. This trajectory propagates a long time on the T_1_ state accompanying with the twists around the C_4_C_5_O_11_H_12_ and C_5_C_4_N_13_O_15_ dihedral angles. At 478.2 fs, it hops to ground S_0_ state. In the overall process, the out-of-plane synchronous motion by O_11_H_12_ vibrating around C_5_O_11_ bond and the nitro group vibrating around C_4_N_13_ bond and this reduces the possibility of hydrogen migration. We show the tunneling hydrogen transfer on T_1_ state for a typical trajectory in Fig. S5 of Supplementary Note 3. This case is similar to non-hydrogen transfer as shown in Fig. 5 except that O_11_H_12_ and O_15_H_12_ bonds oscillate coherently which differs from the non-hydrogen transfer case.

**Figure 6:**
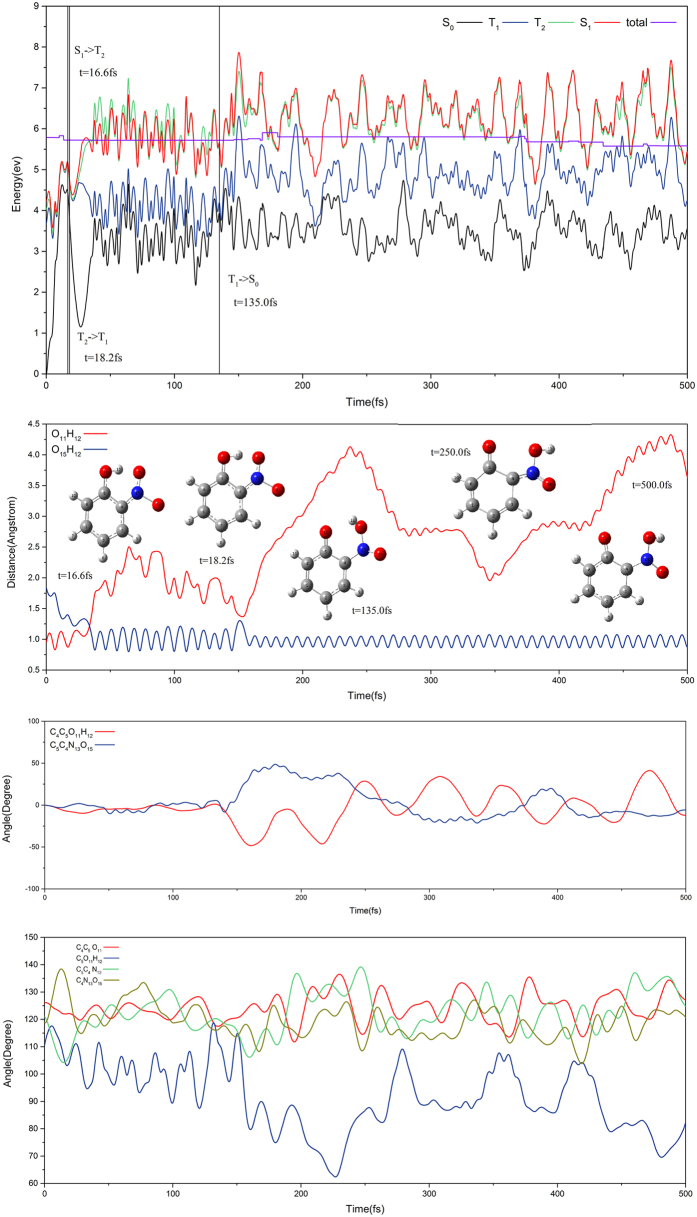
The same as Fig. 5 except for the direct hydrogen transfer trajectory on T_1_ state.

Figure 6 shows the direct hydrogen transfer on T_1_ state for a trajectory that takes the aci-nitrophenol isomerization in the ground state finally. This trajectory hops from the S_1_ to T_2_ state via S_1_T_2_-IC1 at the 16.6 fs and from the T_2_ to T_1_ state via conical intersection at the 18.2 fs. Sequentially, the hydrogen migration is occurred around 36 fs on the T_1_ state accompanied by a shortening of the O_15_H_12_ bond and an elongation of the O_11_H_12_ bond. After 135.0 fs, this trajectory hops from the T_1_ to S_0_ state via S_0_T_1_-IC1. In the initial stage of evolution on the S_0_ state, this trajectory seems attempted back hydrogen transfer, but the reverse process is not successfully taken place. The aci-nitrophenol isomerization reaction appears in the rest of evolution time. The dihedral angle of O_14_N_13_O_15_H_12_ rotates slightly around the C_4_N_13_ bond and the transferred H_12_ turns to away from the original O_11_, finally the aci-isomer2 is observed in this trajectory.

**Figure 7:**
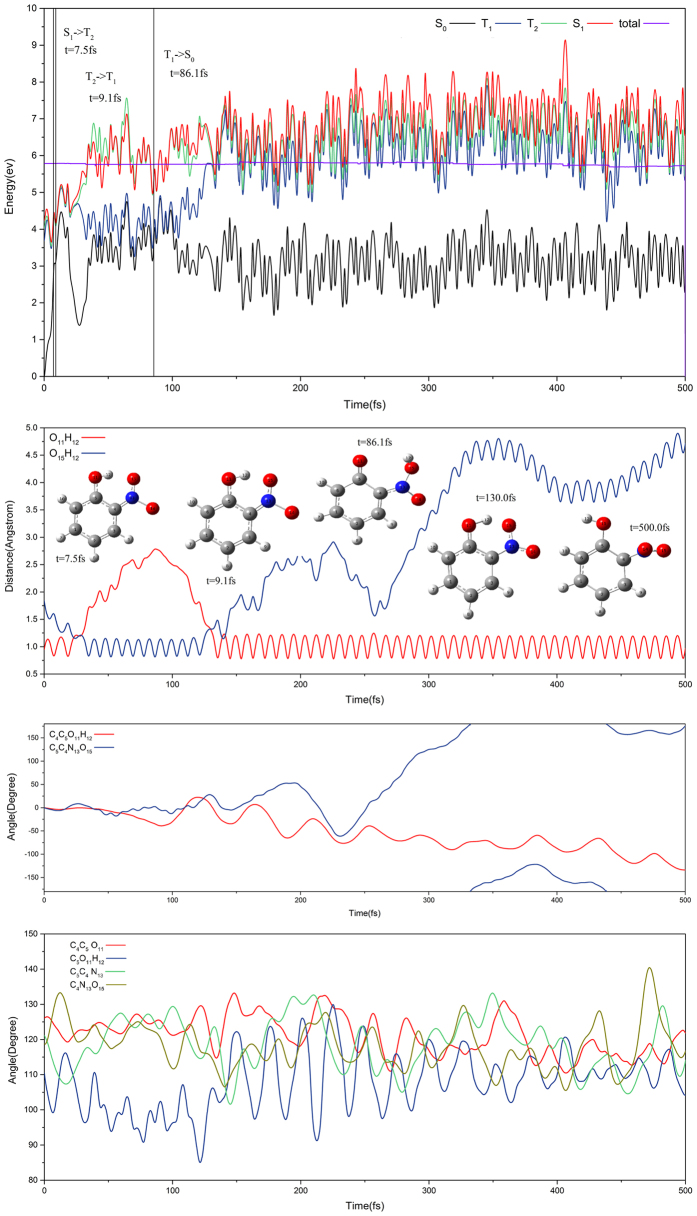
The same as Fig. 5 except for the direct hydrogen transfer trajectory on T_1_ state and back hydrogen transfer on S_0_state.

Figure 7 shows that the trajectory makes the hydrogen transfer on the T_1_ and back hydrogen transfer on S_0_ state. Staring from Frank-Condon region on S_1_ state, the trajectory hops from the S_1_ to T_2_ state via S_1_T_2_-IC1 at the 7.5 fs and from the T_2_ to T_1_ state via conical intersection at the 9.1 fs. Then, the hydrogen transfer is complete and the aci-nitrophenol species is formed at 35 fs on T_1_ state. During trajectory evolution, the distance between O_15_ and H_12_ is shortened gradually until bond forming, and bond angles of C_5_O_11_H_12_ and C_5_C_4_N_13_ change drastically while dihedral angles of C_4_C_5_O_11_H_12_ and C_5_C_4_N_13_O_15_ fluctuate smoothly. At 86.1 fs, trajectory decays to S_0_ state via S_0_T_1_-IC1. Soon after 130.0 fs, the back hydrogen transfer takes place on S_0_ state and regenerates the *o*-nitrophenol. In the rest of evolution, the trajectory shows a periodic fluctuation with H_12_ vibrates around the O_11_H_12_ bond. We have plotted more cases in Supplementary Note 3 for various more complicated fast and slow hydrogen transfer processes.

The present trajectory simulation indicates that the aci-nitro isomers are mostly generated on the triplet states, especially on T_1_ state and this confirms the previous assumption in which the HONO split-off motion is taken place in the triplet manifold^29^,^61^,^62^. The present simulation also indicates that the o-NP dynamic decay via ISC ESIPT is mostly in time scale of subpicosecond (expect tunneling ESIPT) and this is in close agreement with the observations for a large number of nitrated polycyclic aromatic compounds^63^,^64^,^65^. The present simulation shows that there excites the wide Franck-Condon region with very small energy gap between the first excited singlet S_1_ and the second triplet T_2_ states, and this can facilitate the fast ISC radiationless process. Therefore, trajectories hop from S_1_ to T_2_ state along the (O)H-O(NO) barrierless stretching pathway and in the tunneling zone the ESIPT pathways can generally have lower barrier via triplet states than via S_1_ state. The present simulation shows that the (O)H-O(NO) stretch related vibrational modes can enhance ESIPT and the torsion motion of nitro group would hinder the hydrogen transfer reaction.

## Conclusion

**Figure 8:**
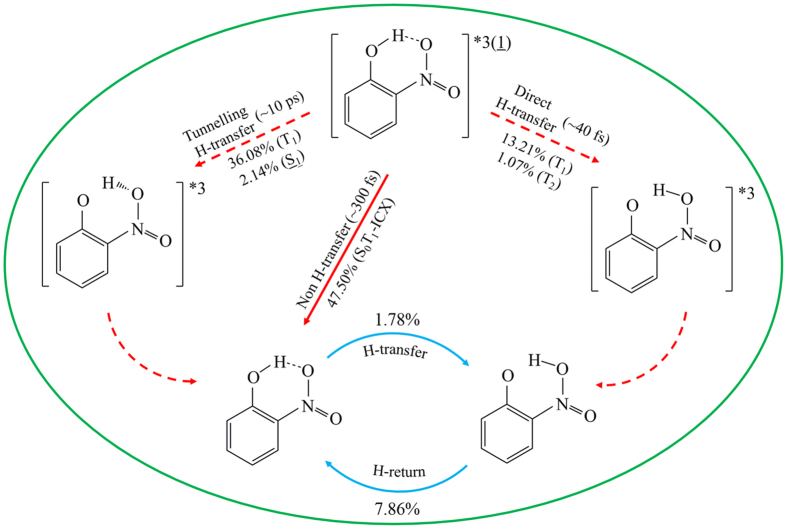
One-slide summary of simulated branch ratios and its time scales for intersystem crossing-branched ESIPT reactions of o-nitrophenol.

By applying ab initio on-the-fly nonadiabatic molecular dynamic simulation for intersystem crossing-branched ESIPT for o-nitrophenol with use of global switching trajectory surface hopping algorithm, we have estimated reaction branch ratios and timescales of various ESIPT processes. As is summarized in Fig. 8, there are three decay branches, the first one is non-hydrogen transfer via newly found S_0_T_1_-ICX with ratio 47.5% and average timescale 300 fs, the second branch is tunneling hydrogen transfer with ratios 36.08% and 2.14% taken place on T_1_ and S_1_ states, respectively and timescale 10 ps, and the third branch is direct hydrogen transfer with ratios 13.21% and 1.07% taken place on T_1_ and T_2_ states, respectively and timescale 40 fs. However, the real lifetimes on excited states from experimental observation might be longer than the present simulated timescales due to some initial extra kinetic energy maybe enhance hydrogen transfer dynamics^29^. Finally, we believe that the present trajectory-based on-the-fly nonadiabatic molecular dynamic simulation method can be generally applied to ultrafast photophysical and photochemical processes in a unified way involved in conical intersections and intersystem crossings for large-scale simulation.

## Additional Information

**How to cite this article**: Xu, C. *et al*. Intersystem crossing-branched excited-state intramolecular proton transfer for o-nitrophenol: An ab initio on-the-fly nonadiabatic molecular dynamic simulation. *Sci. Rep*. **6**, 26768; doi: 10.1038/srep26768 (2016).

## Supplementary Material

Supplementary Information
