# Supplementary material for: Intersystem crossing-branched excited-state intramolecular proton transfer for o-nitrophenol: An ab initio on-the-fly nonadiabatic molecular dynamic simulation
Source: Sci Rep. 2016 May 25;6:26768. doi: 10.1038/srep26768 (PMC4879701; doi:10.1038/srep26768)
Supplement: Supplementary Information [file srep26768-s1.pdf]

Intersystem crossing-branched excited-state intramolecular proton transfer for o-nitrophenol: An ab initio on-the-fly nonadiabatic molecular dynamic simulation

Chao Xu[1], Le Yu[2,3], Chaoyuan Zhu*[2,4], Jianguo Yu[1] & Zexing Cao[4]

[1]College of Chemistry, Beijing Normal University, Beijing 100875, P. R. China

[2]Institute of Molecular Science, Department of Applied Chemistry and Center for Interdisciplinary Molecular Science, National Chiao Tung University, Hsinchu 30010, Taiwan

[3]Key Laboratory of Synthetic and Natural Functional Molecule Chemistry of Ministry of Education, The College of Chemistry &Materials Science, Shaanxi key Laboratory of Physico-Inorganic Chemistry, Northwest University, Xi'an 710069, P. R. China

[4]State Key Laboratory for Physical Chemistry of Solid Surfaces and Fujian Provincial Key Lab of Theoretical and Computational Chemistry, College of Chemistry and Chemical Engineering, Xiamen University, Xiamen 361005, P. R. China.

*E-mail: cyzhu@mail.nctu.edu.tw

Supplementary Information

Chapter **page**

Note 1. Calculating two parameters a^2 and b^2 in equation 4 2

Note 2. Potential energy profiles and key geometries for six ISCs and one CI 3

Note 3. Typical trajectories for ESIPT photoisomerization reactions 8

Note 4. Cartesian coordinates for six ISCs and one CI 15

Note 1. Calculating two parameters a^2 and b^2 in equation 4

Along running trajectory, once we detect hopping point, we can apply multidimensional diabatic forces

$$F_1^{i\alpha} = -\frac{\partial V_1}{\partial R_{i\alpha}} \quad \text{and} \quad F_2^{i\alpha} = -\frac{\partial V_2}{\partial R_{i\alpha}} \tag{S1}$$

where N is number of nuclei in molecule with the its mass m_i (i=1,2,…,N), and $R_{i\alpha}$ stands for x, y, and z component of Cartesian coordinates for the i-th nucleus. Then, we generalize reduced multidimensional force-difference and reduced mass in equation 4 by[37]

$$\frac{|F_2 - F_1|}{\sqrt{\mu}} = \sqrt{\sum_{i=1}^{N} \frac{1}{m_i} \sum_{\alpha=x,y,z} \left(F_2^{i\alpha} - F_1^{i\alpha}\right)^2} \ , \tag{S2}$$

and

$$\frac{\sqrt{|F_2 F_1|}}{\sqrt{\mu}} = \sqrt{\left|\sum_{i=1}^{N} \frac{1}{m_i} \sum_{\alpha=x,y,z} F_2^{i\alpha} F_1^{i\alpha}\right|} \ . \tag{S3}$$

In the case of intersystem crossing, this can be done straightforward at hopping point where $d(t) = |U_2(t) - U_1(t)| / |V_2(t) - V_1(t)|$ is a local maximum, and diabatic force in equation S1 can be directly obtained from ab initio quantum chemistry calculation where diabatic forces are just given (one is singlet-state potential surface and another is triplet potential energy surface). Diabatic coupling in equation 4 is given by V_{12} = spin-orbital coupling.

However, in the case of conical intersection, the ab initio quantum chemistry calculation only gives adiabatic forces, we need generalize diabatic forces at minimum separation between two adiabatic potential energy surfaces. Along running trajectory, we

have three consecutive time steps ($t_1 < t_2 < t_3$) where adiabatic gap $\left|U_+(t_2) - U_-(t_1)\right|$ is a local minimum. We simply make one linear connection of upper adiabatic force at t_1 with lower adiabatic force at t_3, and another linear connection of lower adiabatic force at t_1 with upper adiabatic force at t_3, namely[37]

$$F_1^{i\alpha}(t_2) = -\frac{\partial V_1}{\partial R_{i\alpha}(t_2)} = \frac{-1}{R_{i\alpha}(t_3) - R_{i\alpha}(t_1)} \times \left[\frac{\partial U_-}{\partial R_{i\alpha}(t_3)}\left(R_{i\alpha}(t_2) - R_{i\alpha}(t_1)\right) - \frac{\partial U_+}{\partial R_{i\alpha}(t_1)}\left(R_{i\alpha}(t_2) - R_{i\alpha}(t_3)\right)\right] \tag{S4}$$

and

$$F_2^{i\alpha}(t_2) = -\frac{\partial V_2}{\partial R_{i\alpha}(t_2)} = \frac{-1}{R_{i\alpha}(t_3) - R_{i\alpha}(t_1)} \times \left[\frac{\partial U_+}{\partial R_{i\alpha}(t_3)}\left(R_{i\alpha}(t_2) - R_{i\alpha}(t_1)\right) - \frac{\partial U_-}{\partial R_{i\alpha}(t_1)}\left(R_{i\alpha}(t_2) - R_{i\alpha}(t_3)\right)\right], \tag{S5}$$

where U_+ and U_- are upper and lower adiabatic potential energy surfaces, respectively along a trajectory. Diabatic coupling in equation 4 is given by $2V_{12}(t_2) = U_+(t_2) - U_-(t_2)$. At hopping point, if attempted hopping is successfully achieved, we can define hopping direction from diabatic forces[37]

$$s_{i\alpha} = \frac{F_2^{i\alpha} - F_1^{i\alpha}}{\sqrt{m_i}} \tag{S6}$$

with normalized vector as

$$\mathbf{n}_i = \frac{1}{\sqrt{s_{ix}^2 + s_{iy}^2 + s_{iz}^2}}\left(s_{ix}, s_{iy}, s_{iz}\right). \tag{S7}$$

Note 2. Potential energy profiles and key geometries for six ISCs and one CI

We have plotted vertical excitation energies of nitro and aci-nitro isomers at all

stationary points of the S_0 states optimized at 6SA-CASSCF (10, 10) /6-31G (d, p) level in Fig.S1(a) and with MRCI+Q/cc-pVDZ energy corrections in Fig.S1(b). We have plotted adiabatic energy profiles for all nitro and aci-nitro isomers, transition states, and intersystem crossings on four electronic states optimized at 6SA-CASSCF (10, 10) /6-31G (d, p) level in Fig.S2(a) and with MRCI+Q/cc-pVDZ energy corrections in Fig.S2(b). Figures S1 and S2 show that energy orders within four states are well preserved between CASSCF and MACI calculations and the relative energy differences for most of these geometries are smaller than 0.1 eV and relative gap differences at intersystem crossings are even smaller as shown in Table S1.

Table S1: Energy differences kcal/mol (eV) at six ISCs and one CI optimized at 6SA-CASSCF (10, 10) /6-31G (d, p) level and corrected by MRCI/cc-pVDZ.

ISC(CI)-Geometry	Method	S_0 or S_1	T_1 or T_2 or S_1
S_0T_1-ICX	CASSCF	70.6(3.06)	70.6(3.06)
	MRCI	65.9(2.86)	68.3(2.96)
S_0T_1-IC1	CASSCF	41.1(1.78)	41.1(1.78)
	MRCI	48.0(2.08)	48.2(2.09)
S_0T_1-IC2	CASSCF	74.2(3.22)	74.2(3.22)
	MRCI	72.6(3.15)	73.2(3.18)
S_1T_1-IC	CASSCF	97.2(4.22)	97.2(4.22)
	MRCI	98.0(4.25)	100.8(4.37)
S_1T_2-IC1	CASSCF	87.0(3.77)	87.0(3.77)
	MRCI	95.1(4.12)	90.5(3.92)
S_1T_2-IC2	CASSCF	61.4(2.66)	61.4(2.66)
	MRCI	53.7(2.33)	53.8(2.33)
S_0S_1-CI	CASSCF	69.7(3.02)	69.7(3.02)
	MRCI	69.1(3.00)	71.1(3.08)

We present key geometry changes for six intersystem crossings and one conical intersection in Fig.S3, and we can see that newly found S_0T_1-ICX is close Franck-Condon geometry and the conical intersection S_0S_1-CI is close to S_0T_1-IC1 geometry which all belong to geometry configuration after hydrogen transfer.

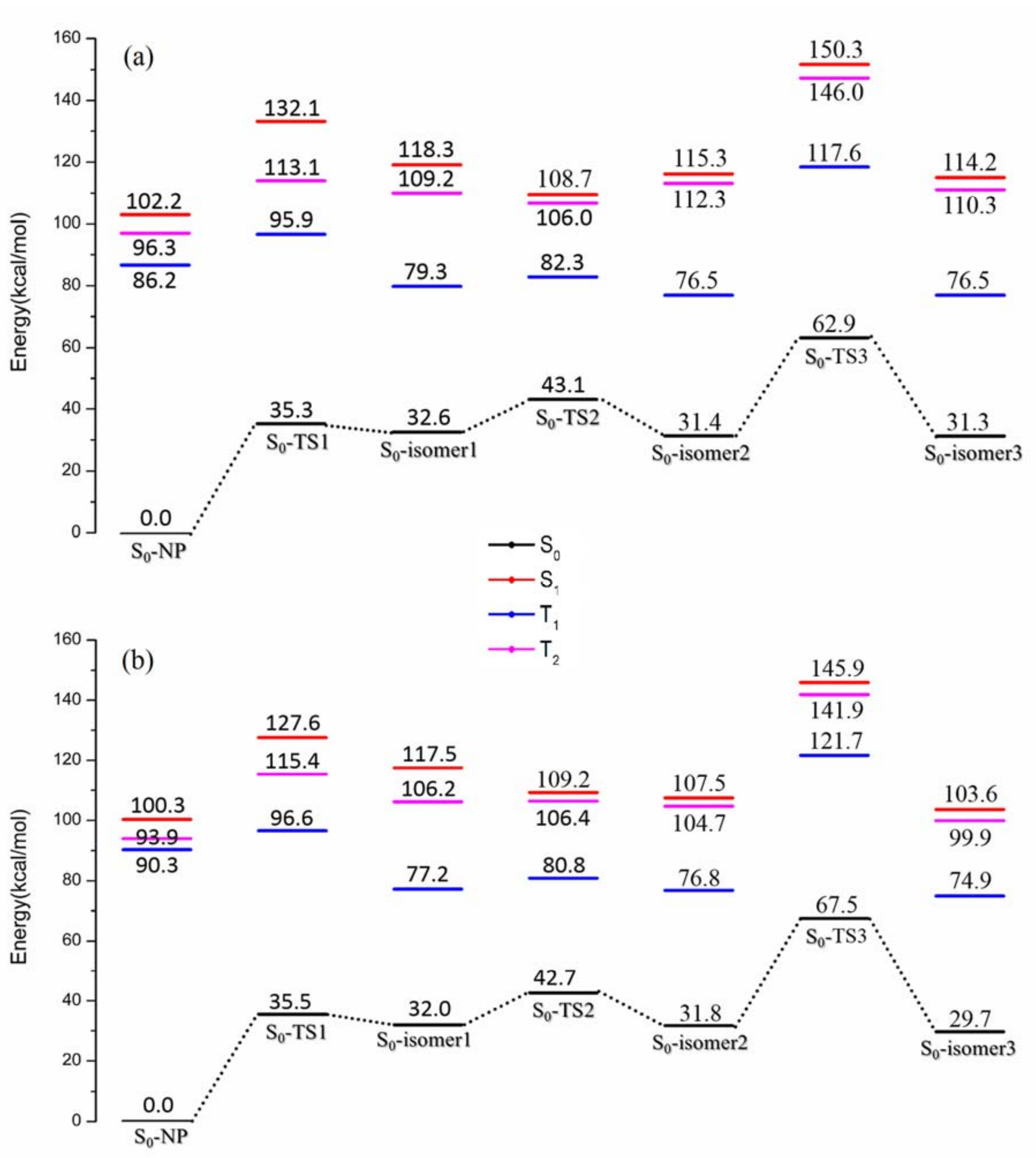

Figure S1| Vertical excitation energies of nitro and aci-nitro isomers at all stationary points of the S_0 state optimized at 6SA-CASSCF (10, 10) /6-31G (d, p) level. **(a)** CASSCF energies and **(b)** MRCI corrections

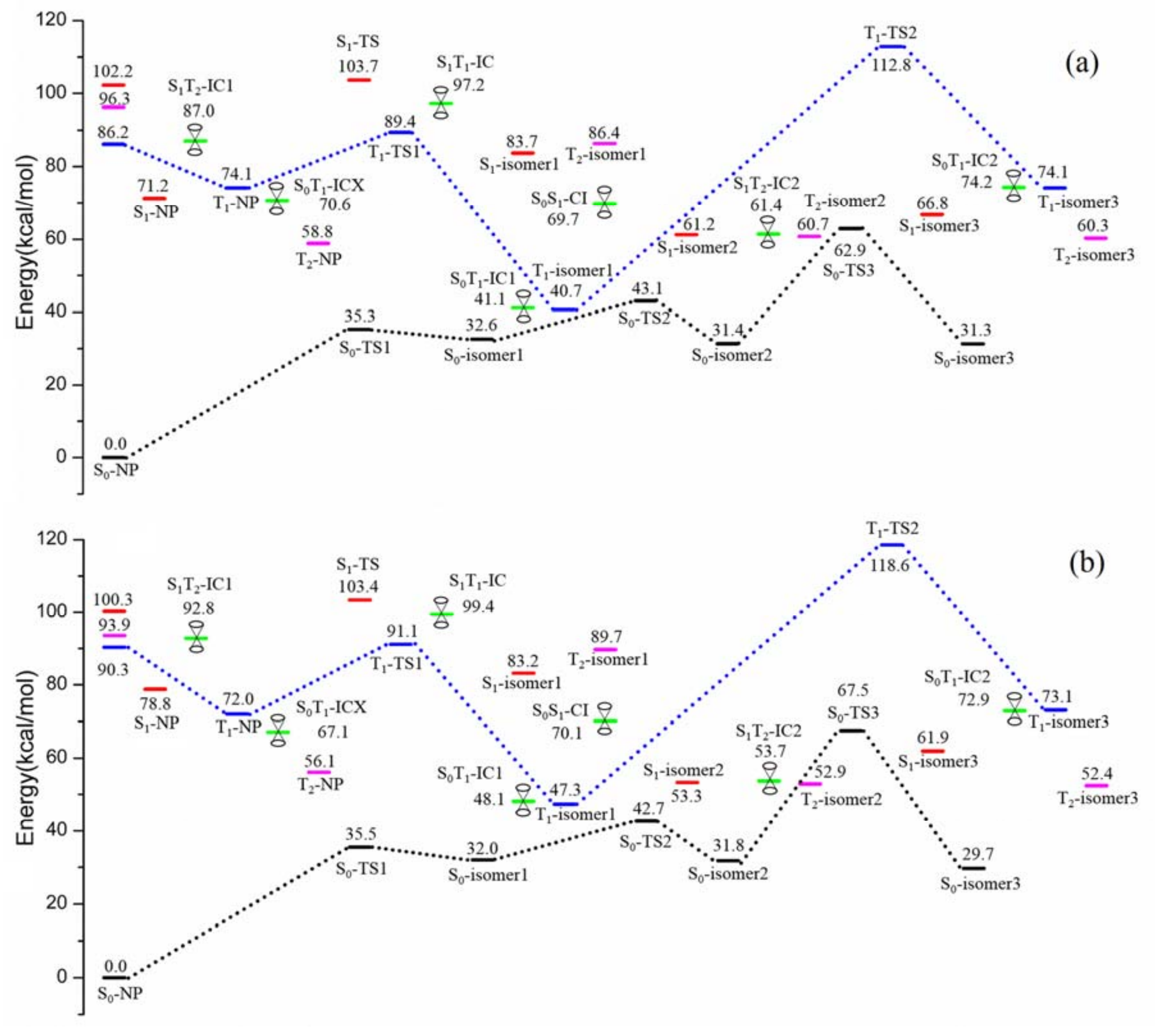

Figure S2| Potential energies profiles for all nitro and aci-nitro isomers, transition states, intersystem crossings and conical intersection on the S_0, T_1, T_2 and S_1 states optimized at 6SA-CASSCF (10, 10) /6-31G (d, p) level. **(a)** CASSCF energies and **(b)** MRCI energy corrections.

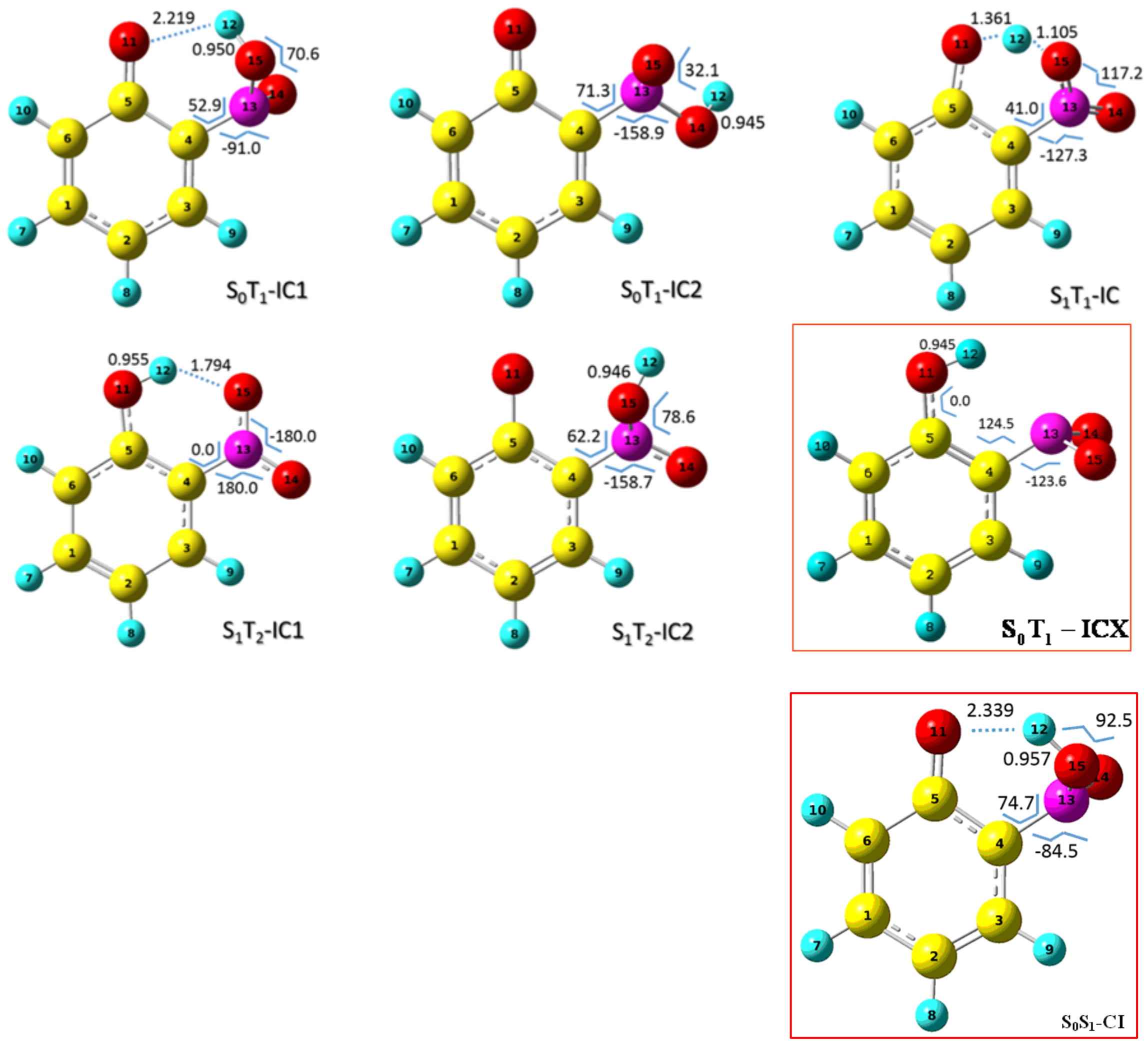

Figure S3| Key geometries of the six ISCs and one CI optimized at 6SA-CASSCF (10, 10) /6-31G (d, p) level (5 of six are found in the previous paper: J. Phys. Chem. A 2015, 119, 10441−10450. New one S_0T_1-ICX is found by the present nonadiabatic molecular dynamic simulation)

Note 3. Typical trajectories for ESIPT photoisomerization reactions

We show some typical trajectories for various ESIPT photoisomerization to explore the aci-nitrophenol or back to the staring o-nitrophenol.

Figure S4 shows that the trajectory makes the hydrogen transfer on the S_0 state. Staring from Frank-Condon region on S_1 state, the trajectory hops from the S_1 to T_2 state via S_1T_2–IC1 at the 7.3 fs, from the T_2 to T_1 state via conical intersection at the 14.2 fs and from the T_1 to S_0 state via S_0T_1–ICX at the 41.6 fs. Then, the trajectory propagates in the S_0 state along with periodic stretch of $O_{11}H_{12}$ bond plus nonsymmetrical motions of four bond angles ($C_4C_5O_{11}$, $C_5O_{11}H_{12}$, $C_5C_4N_{13}$ and $C_4N_{13}O_{15}$). At the same time, $C_4C_5O_{11}H_{12}$ and $C_5C_4N_{13}O_{15}$ dihedral angles are vibrating with small amplitude. After 230.0 fs, the hydrogen transfer is triggered by accompanying with decrease of bond angle $C_4C_5O_{11}$ and synchronous motion of bond angle $C_5C_4N_{13}$. Interestingly, about 350.0 fs, the $O_{11}H_{12}$ and $O_{15}H_{12}$ distances are almost equal, but the back hydrogen transfer is not observed because of the large distance of O_{11} and N_{13}.

Figure S5 shows that the trajectory makes the tunneling hydrogen transfer on the T_1 state.

Figure S6 shows that the trajectory makes the hydrogen transfer on the T_1 state and then running back and forth between the aci-isomer1 and aci-isomer2 on S_0 state. Staring from Frank-Condon region on S_1 state, the trajectory hops from the S_1 to T_2 state via S_1T_2–IC1 at the 7.7 fs and from the T_2 to T_1 state via conical intersection at the 13.9 fs. Then, the hydrogen transfer is taken place at 25 fs on T_1 state. Finally, it hops to the aci-isomer2 of S_0 state via S_0T_1-IC1 at 61.3 fs. During the rest of evolution, it runs aci-nitrophenol tautomerization reaction back and forth between the aci-isomer1 and aci-

isomer2 on S_0 state.

Figure S7 shows that following consecutive processes: S_1 (FC-*o*-nitrophenol) → S_1T_2 –IC1 (at 7.4 fs) → T_2 → T_2T_1-IC (at 9.2 fs)→ T_1 → aci-isomer1 (slow hydrogen transfer on T_1) → S_0T_1 –IC1 (303.5 fs)→ aci-somer2 (on S_0) → aci-somer1(on S_0) → aci-somer2(on S_0). Unlike previous trajectory as shown in Figure S6, the hydrogen transfer does not take place on the T_1 state immediately, the H_{12} and O_{15} (O_{11}) distance oscillates decrease (increase) and increase (decrease) for a long time, the hydrogen transfer is observed until about 270.0 fs. Once upon to the ground S_0 state, dihedral angles $C_4C_5O_{11}H_{12}$ and synchronous $C_5C_4N_{13}O_{15}$ motions dominate the remaining processes, the trajectory runs aci-nitrophenol tautomerization reaction back and forth between the aci-isomer1 and aci-isomer2 on S_0 state.

Figure S8 shows that following consecutive processes: the trajectory finishes S_1→T_2→T_1 processes with passing S_1T_2 –IC1 at 9.0 fs and T_2T_1-IC at 13.3 fs, and then completes the hydrogen transfer quickly and decays to ground at 89.3 fs. Afterwards, the trajectory roams accompanied by rotation of H_{12} around the $O_{15}H_{12}$ bond. On S_0 state, the trajectory runs back and forth between aci-isomer1 and aci-isomer2 for a while. Finally back hydrogen transfer takes place with leading to formation of the initial *o*-nitrophenol.

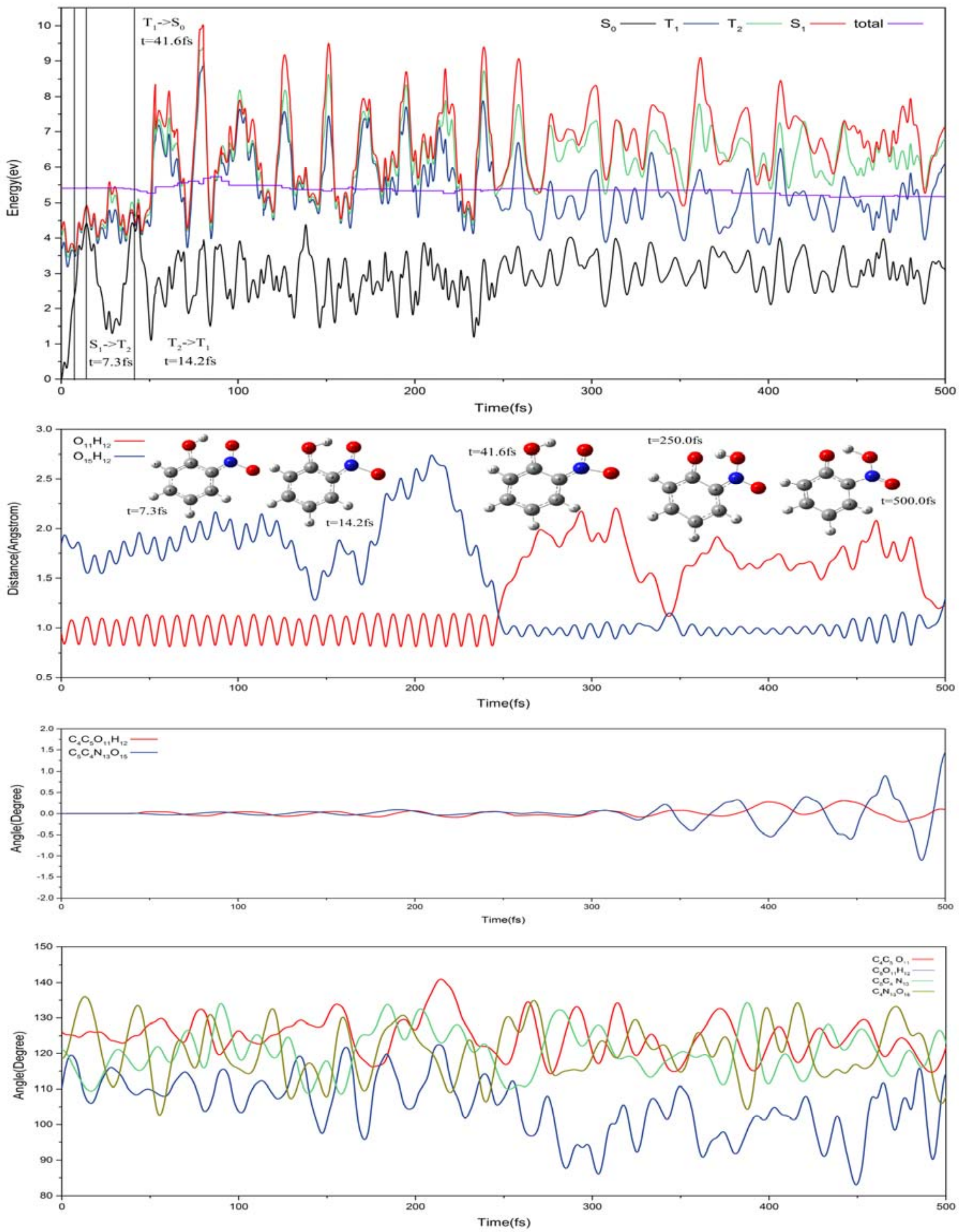

Figure S4| This is case for hydrogen transfer taking place on S_0 state. The first panel is for potential energy profiles. The second pane is evolution for bond lengths $O_{11}H_{12}$ and $O_{15}H_{12}$, the third is for dihedral angles $C_4C_5O_{11}H_{12}$ and $C_5C_4N_{13}O_{15}$ and the fourth is for bond angles $C_4C_5O_{11}$, $C_5O_{11}H_{12}$, $C_5C_4N_{13}$, and $C_4N_{13}O_{15}$.

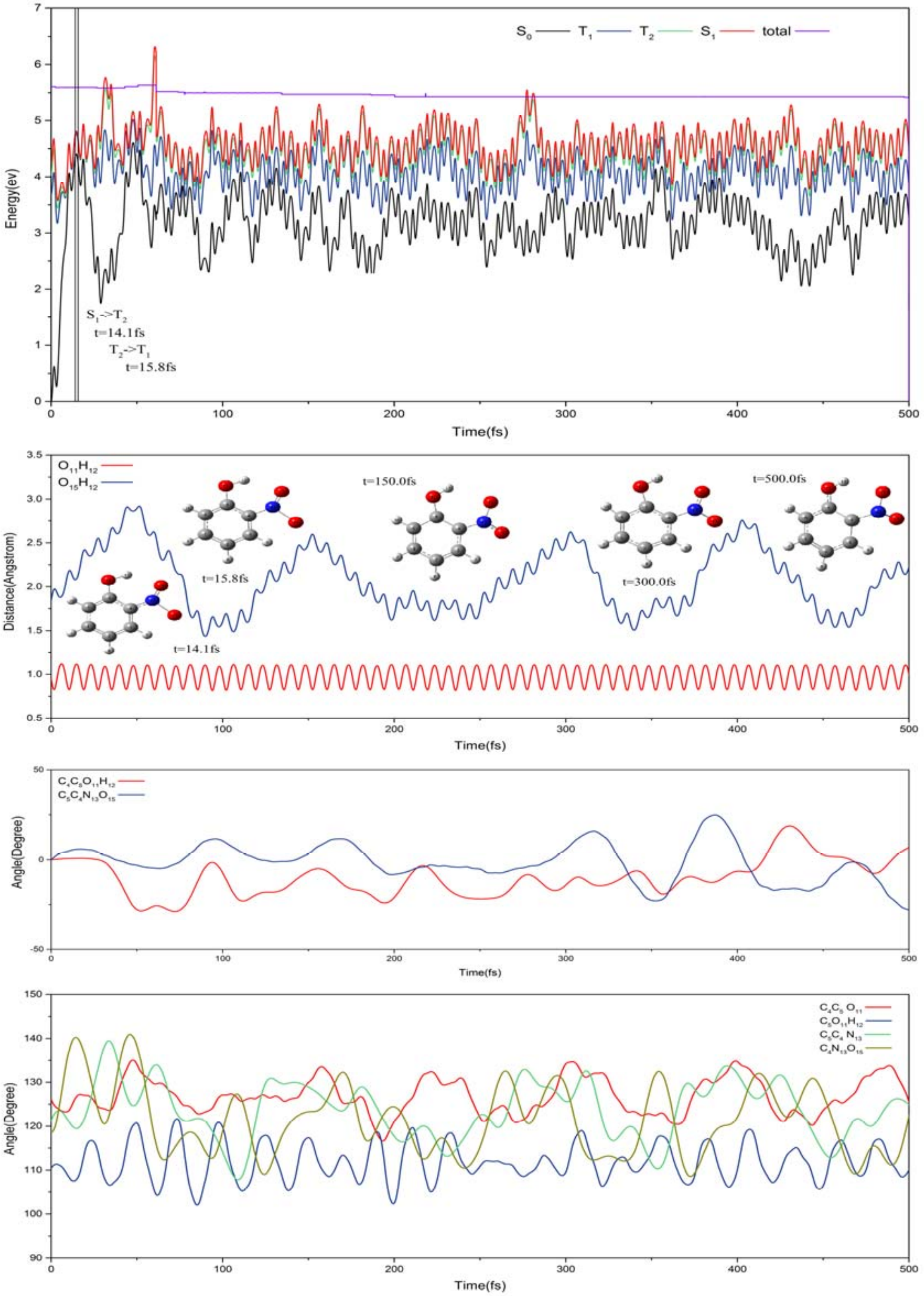

Figure S5| The same as Figure S4 except for the case of tunneling hydrogen transfer taking place on T_1 state.

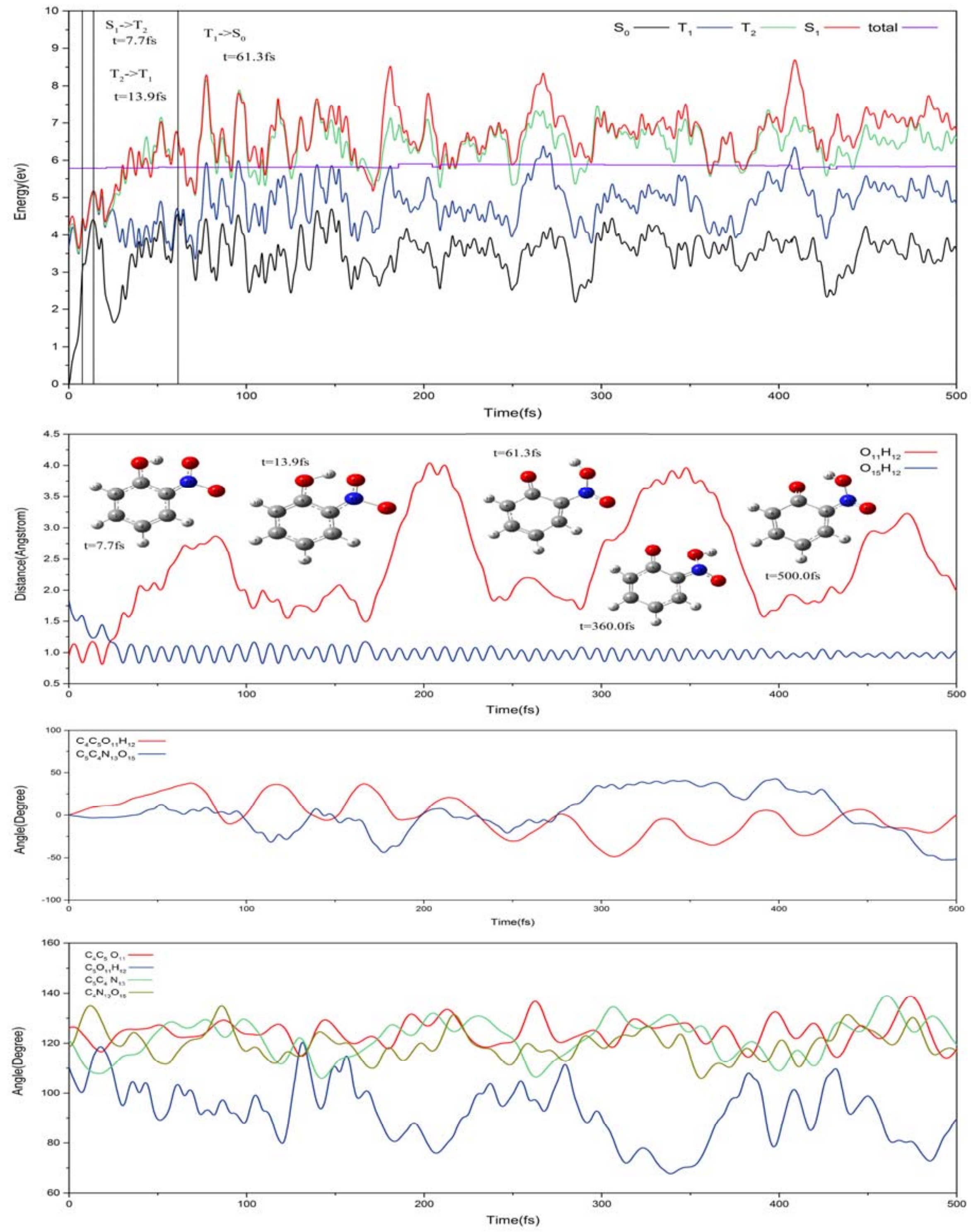

Figure S6| The same as Figure S4 except for the case of fast hydrogen transfer taking place on T_1 state and then running back and forth between the aci-isomer1 and aci-isomer2 on S_0 state.

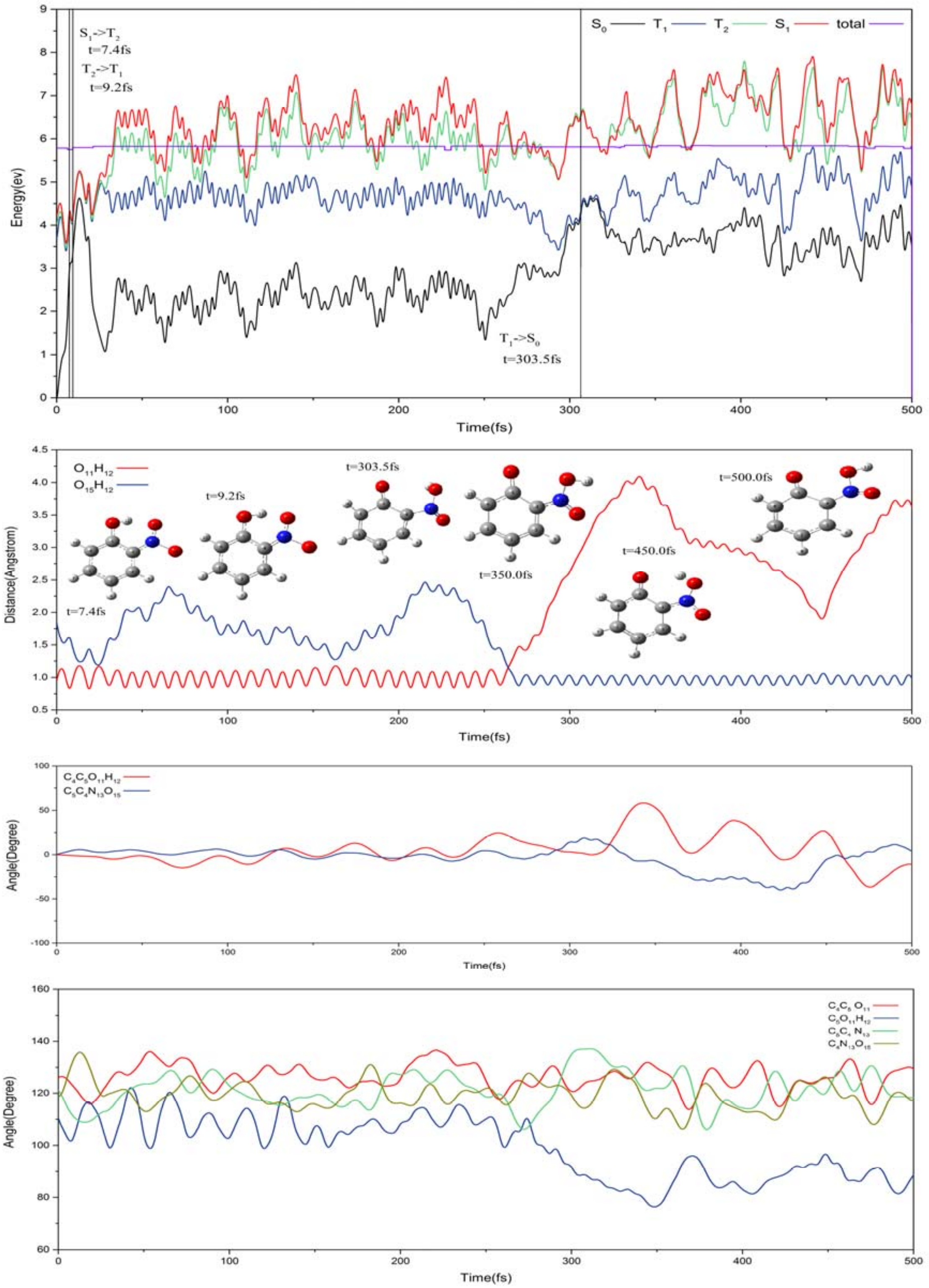

Figure S7| The same as Figure S4 except for the case of slow hydrogen transfer taking place on T_1 state and then running back and forth between the aci-isomer1 and aci-isomer2 on S_0 state.

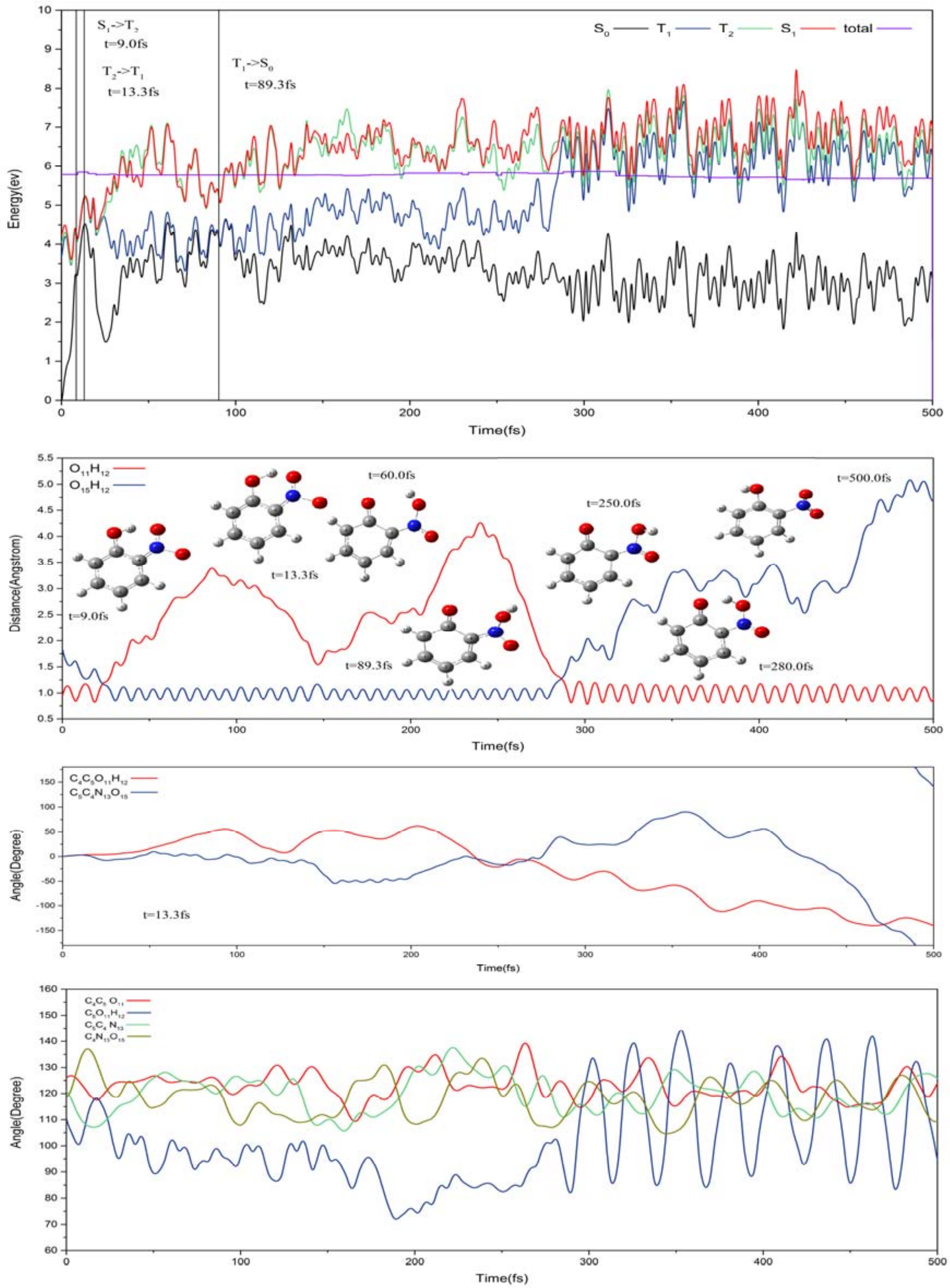

Figure S8| The same as Figure S4 except for the case of fast hydrogen transfer taking place on T_1 state and then running back and forth between the aci-isomer1 and aci-isomer2 on S_0 state for a while. Finally, back hydrogen transfer takes place on S_0 state.

Note 4. Table S2: Cartesian coordinates (in angstrom) of all optimized geometries for six intersystem crossings and one conical intersection between singlet and triplet states optimized at the 6SA-CASSCF (10, 10) /6-31G (d, p) level.

Table S2.1 : S_0T_1-ICX (newly founded)

	X	Y	Z
C1	0.24025	-0.16394	-2.57481
C2	1.39557	-0.45109	-1.82461
C3	1.34563	-0.36670	-0.44951
C4	0.14614	0.00347	0.18177
C5	-0.98138	0.28356	-0.54348
C6	-0.93407	0.19873	-1.94791
H7	0.26940	-0.22694	-3.64785
H8	2.30703	-0.73303	-2.31878
H9	2.20948	-0.57919	0.15271
H10	-1.82657	0.42166	-2.50184
O11	-2.15141	0.64058	0.00968
H12	-2.07945	0.66746	0.95147
N13	0.04411	0.10619	1.61494
O14	0.91933	0.93423	2.17477
O15	0.39383	-0.99359	2.27388

Table S2.2 : S_0T_1-IC1

	X	Y	Z
C1	0.02190	0.00396	-2.65251
C2	1.30769	-0.13517	-2.08266
C3	1.46976	-0.17933	-0.67856
C4	0.37717	-0.08110	0.14552
C5	-0.97319	0.08756	-0.39913
C6	-1.09159	0.10994	-1.85004
H7	-0.08244	0.02887	-3.72197
H8	2.17002	-0.21535	-2.71751
H9	2.44647	-0.28635	-0.24512
H10	-2.07982	0.22082	-2.25564
O11	-1.95030	0.21583	0.33609
H12	-1.11766	-0.58908	2.22861
N13	0.55252	-0.07822	1.56007
O14	0.78945	1.00850	2.12864
O15	-0.24165	-0.95713	2.23574

Table S2.3: S_0T_1-IC2

	X	Y	Z
C1	0.46375	−0.10810	−2.58859
C2	1.58959	−0.14770	−1.72929
C3	1.43108	−0.07066	−0.32479
C4	0.17948	0.06286	0.22002
C5	−1.02860	0.14271	−0.62699
C6	−0.79950	0.02061	−2.07187
H7	0.60843	−0.17789	−3.65155
H8	2.57647	−0.24094	−2.14237
H9	2.29013	−0.10070	0.31612
H10	−1.67406	0.06138	−2.69335
O11	−2.12384	0.31951	−0.16918
H12	0.79838	1.20106	2.89729
N13	−0.04559	0.12768	1.62906
O14	−0.58880	−0.91488	2.10422
O15	1.08464	0.51208	2.31727

Table S2.4: S_1T_2-IC1

	X	Y	Z
C1	0.23347	0.00695	−2.67909
C2	1.44306	0.00214	−2.00306
C3	1.47247	0.00058	−0.52981
C4	0.26548	0.00008	0.22781
C5	−0.97687	0.00029	−0.46491
C6	−0.99725	0.00069	−1.90489
H7	0.18927	0.00072	−3.75044
H8	2.38009	0.00012	−2.52495
H9	2.40914	0.00063	−0.01392
H10	−1.96033	0.00005	−2.37435
O11	−2.15824	0.00061	0.12511
H12	−2.03765	0.00082	1.07251
N13	0.33122	0.00071	1.63313
O14	1.43936	0.00005	2.16039
O15	−0.71436	0.00016	2.28376

Table S2.5: S_1T_2-IC2

	X	Y	Z
C1	0.24468	−0.11354	−2.60474
C2	1.45280	−0.16821	−1.89086
C3	1.44904	−0.08077	−0.52321
C4	0.24575	0.06541	0.16206
C5	−0.94667	0.13552	−0.54849
C6	−0.95599	0.04038	−1.93879
H7	0.25086	−0.18081	−3.67738
H8	2.38257	−0.28129	−2.41748
H9	2.36237	−0.11359	0.03895
H10	−1.88789	0.09815	−2.46998
O11	−2.13969	0.30639	0.12242
H12	−1.00875	−0.43502	2.86533
N13	0.20521	0.18271	1.58491
O14	1.23036	0.51885	2.19866
O15	−0.52085	−0.83670	2.16129

Table S2.6: S_1T_1-IC1

	X	Y	Z
C1	0.05013	−0.03161	−2.64108
C2	1.28831	−0.14882	−2.08654
C3	1.44310	−0.14952	−0.62841
C4	0.37729	0.05096	0.10717
C5	−0.94509	0.15282	−0.43379
C6	−1.10593	0.10024	−1.82004
H7	−0.06150	−0.02302	−3.70996
H8	2.17160	−0.20712	−2.69740
H9	2.40055	−0.30036	−0.16347
H10	−2.08139	0.23673	−2.25298
O11	−1.90274	0.29126	0.48315
H12	−1.36651	−0.41529	1.51601
N13	0.42971	−0.06540	1.56496
O14	1.10365	0.66563	2.22293
O15	−0.47570	−0.83549	2.01663

Table S2.7: S_0S_1-CI

	X	Y	Z
C1	2.61303	−0.14070	0.05989
C2	1.94117	−1.38760	−0.21422
C3	0.58794	−1.45075	−0.19832
C4	−0.13183	−0.28719	0.11377
C5	0.46412	1.01074	0.30587
C6	1.92613	1.00653	0.31346
H7	3.68839	−0.12584	0.05378
H8	2.52470	−2.26465	−0.42330
H9	0.06442	−2.36916	−0.39233
H10	2.41968	1.94042	0.50774
O11	−0.22865	2.01142	0.38774
H12	−1.97977	1.18547	−0.92424
N13	−1.58603	−0.25012	0.14754
O14	−2.20235	−0.29582	1.19121
O15	−2.20935	0.25615	−0.92934
